# Recombination, mobile genetic elements, and genetic transfer contribute to the adaptation of *Streptococcus uberis* causing mastitis

**DOI:** 10.1186/s13567-026-01795-x

**Published:** 2026-07-07

**Authors:** Anyaphat Srithanasuwan, Yang Zou, Ruth N. Zadoks, Witaya Suriyasathaporn, Ynte H. Schukken

**Affiliations:** 1 Infectious Disease Epidemiology, Wageningen University, Droevendaalsesteeg 1, 6708PB Wageningen, The Netherlands; 2 School of Veterinary Medicine, Faculty of Veterinary Medicine, Chiang Mai University, Chiang Mai, 50100 Thailand; 3 Sydney School of Veterinary Science, Faculty of Science, University of Sydney, Camden, NSW 2570 Australia; 4 Research Center of Producing and Development of Products and Innovations for Animal Health and Production, Chiang Mai University, Chiang Mai, 50100 Thailand; 5 Cambodia Campus, Asian Satellite Campuses Institute, Nagoya University, Nagoya, 464-8601 Japan; 6 GD Animal Health, PO Box 9, 7400 AA Deventer, The Netherlands; 7 Department of Population Health Sciences, Utrecht University, 3584 CL Utrecht, The Netherlands

**Keywords:** *Streptococcus uberis*, mobile genetic elements, antimicrobial resistance, recombinant event, prophage

## Abstract

*Streptococcus uberis* is a major cause of bovine mastitis. However, the genomic mechanisms that facilitate adaptation of the pathogen within different host-associated environment or selection pressures remain poorly understood. This study analyzed whole-genome sequence data from three Thai dairy herds to investigate the contributions of recombination and mobile genetic elements (MGEs) to *S. uberis* evolution and adaptation. Among the 138 *S. uberis* genomes, 42 core genome sequence types (cgSTs) were identified, along with frequent detection of MGEs such as plasmid-associated genes (81.1% of isolates), prophages (67.4% of isolates), and insertion sequences (26.1% of isolates). The isolates from farm A exhibited the longest recombined fragment size, but with extremely low recombination frequency and recombination-to-mutation ratio. By contrast, the isolates from farm B, which had the highest prevalence of antimicrobial resistance (AMR) gene, showed a high recombination-to-mutation ratio (*R*/*θ* = 4.42) and more frequently contained MGEs associated with AMR genes. Finally, isolates from farm C shared a single core genome and AMR profile but harbored diverse prophages. Several prophages shared high sequence similarity (>99%) with phages infecting other bacterial genera, suggesting that ecological overlap between bacterial species may facilitate cross-genus genetic exchange, highlighting the influence of microbial ecology on the evolution of *S. uberis*. Collectively, our results illustrate the variety of mechanisms and genetic elements that contribute to the adaptive evolution of *S. uberis* in dairy farming environments.

**Supplementary Information:**

The online version contains supplementary material available at 10.1186/s13567-026-01795-x.

## Introduction

Mastitis causes significant economic losses in dairy farming worldwide, resulting in reduced milk production and quality, increased treatment costs, and the culling of animals suffering from severe clinical disease or chronic infections. *Streptococcus uberis* is one of the most common intramammary pathogens, causing both clinical and subclinical bovine mastitis, resulting in considerable financial losses [1]. *Streptococcus uberis* is classified as both an environmental and a contagious pathogen, capable of surviving in diverse environments, including the gastrointestinal tract (rumen and gut), feces, bedding, pasture, cow skin, and the mammary gland [2, 3]. *S. uberis* is a significant cause of subclinical mastitis in a variety of high-income production settings [4–6] as well as low- or middle-income countries [7, 8]. Whole-genome sequencing has revealed that *S. uberis* exhibits substantial genetic diversity, reflecting its ability to survive across multiple ecological niches [9, 10]. This diversity can be driven by mutation, recombination, and the acquisition of foreign DNA through mobile genetic elements (MGEs), enabling *S. uberis* to respond rapidly to selective pressures such as host immunity, antimicrobial use, and environmental conditions.

Antimicrobial resistance (AMR) can be disseminated by MGEs, such as plasmids, transposons, insertion sequences, or bacteriophages [11, 12]. Horizontal gene transfer can occur within species or between species, as described for integrative and conjugative elements (ICEs) in mastitis-causing *Streptococcus* species, including *S. uberis* [13], and for plasmids in streptococcal species that share a host-species or body system as their ecosystem [14]. While MGEs and recombination are known to contribute to the genomic evolution of *S. uberis* in small-scale dairy herds [15], little is known about how these processes manifest in different farming contexts or under high selection pressure for AMR, as commonly observed in dairy farms in tropical areas, including Thailand [16]. In addition, little is known about phages in *S. uberis*, even though dedicated phage typing schemes have been developed for another mastitis-causing pyogenic *Streptococcus,* i.e., *Streptococcus agalactiae* [17, 18].

Therefore, this study investigated the role of recombination, MGEs, and their association with AMR genes in *S. uberis* adaptive evolution. Through whole-genome sequencing and comparative genomic analyses, this study examined the recombination events and MGEs associated with horizontal transfer of AMR genes in *S. uberis* isolates from Thailand and investigated how MGEs, particularly prophages, contribute to the evolutionary diversity and adaptation of *S. uberis*.

## Materials and methods

### Isolate and herd data

A total of 138 *S. uberis* whole-genome sequences from three dairy herds in Thailand were generated, with details of bacterial selection, preparation, and genome sequencing provided in a previous publication [7]. The sequenced isolates represented 101 episodes of intramammary infection (IMI) in 99 quarters of 45 cows. The definition of IMI followed a previous study [19], in which a quarter was considered to have an IMI when >1000 CFU/mL of the pathogen was cultured from a single sample. Details on cow-quarter, sampling time, farm characteristics, mastitis control practices, and AMU have also been described previously [7]. Briefly, *S. uberis* isolates were obtained from different farms in different years: farm A (2013, *n* = 29), farm B (2019, *n* = 53), and farm C (2023, *n* = 56). All the participating farms exhibited suboptimal husbandry and milking hygiene, including a lack of routine maintenance of milking equipment and poor mastitis control policies. Antimicrobial usage was reported to be highest in farm A, followed by farm B and farm C. The decision to administer antibiotics varied by farm and was made by workers (farm A), the owner (farm B), or veterinarians (farm C), with suboptimal dosing when decisions were not made by veterinarians.

To quantify within-farm diversity of the *S. uberis* population, Simpson’s index of discrimination (SID) was calculated on the basis of sequence type (ST), AMR patterns and combined AMR–MGE patterns. The SID ranges from 0 to 1, where 0 indicates no discrimination (all isolates within the farm share the same profile), and a value of 1 indicates maximum discrimination (each isolate exhibits a unique profile).

### Core genome and multilocus sequence typing

Core genome multilocus sequence typing (cgMLST) and multilocus sequence typing (MLST) were performed via the PubMLST web server (accessed March 14, 2025) [20]. Allelic profiles were matched against the PubMLST database to determine sequence types (STs) and core genome sequence types (cgSTs). Minimum-spanning trees were generated on the basis of both the cgMLST and MLST schemes with all *S. uberis* global genomes in the pubMLST database, including the Thai *S. uberis* genomes from the present study. All genomes were annotated at the defined loci via successive rounds of automated Bacterial Isolate Genome Sequence Database (BIGSdb) allele assignment using the default identity and alignment thresholds. Gene sequences were compared against curated reference alleles to assign allele numbers and determine sequence types (STs) on the basis of MLST profiles. Phylogenetic relationships and cluster visualization were produced with GrapeTree via the built-in BIGSdb GrapeTree tool [21].

### Detection of recombination

All genome assemblies were screened and extracted from a core genome alignment generated using Roary with SNP-sites v2.5.1 [22]. The resulting core single nucleotide polymorphism (SNP) alignment was used for subsequent recombination analyses. Recombination within the core genome was assessed using ClonalFrameML [23], using default parameters. The estimated parameters included the recombination-to-mutation ratio (*R*/*θ*), the inverse of the mean recombination tract length (1/*δ*), the average size of recombined fragments (*I*), and the divergence of imported DNA (*D*), providing an overview of the scale and impact of recombination across the dataset. To visualize potential nontree-like evolutionary signals associated with recombination, relationships among isolates were visualized using neighbor-net networks generated in SplitsTree v6.5.1 [24]. This method produces split graphs that reveal conflicting phylogenetic signals, offering complementary insight to the model-based estimates obtained from ClonalFrameML, which assume bifurcating evolution.

### Identification of mobile genetic elements and antimicrobial resistant genes

Mobile genetic elements and AMR determinants were characterized using a combination of reference-based annotation tools and previously curated datasets. MGEs and AMR genes were screened by Mobile Element Finder v1.0.3 (accessed March 13, 2025) [25], using default parameters. Because the study relied on short-read Illumina assemblies, plasmid-associated genes could be identified but the presence of actual plasmids was not evaluated. Prophage regions were identified across all isolates using the PHASTEST web server (accessed March 27, 2025) [26]. Prophage regions from each prophage type were extracted, and the genomic similarity among prophage types was visualized using Genofig [27]. Prophages and their host bacteria were identified by Phagonaute [28] and Virus-Host DB [29] (accessed 8 January 2026). For AMR gene characterization, resistance determinants that were detected using Mobile Element Finder were supplemented with the AMR gene data previously reported for this isolate collection [7]. Combining both sources ensured comprehensive coverage of acquired AMR genes and MGEs carrying resistance determinants.

## Results

### Core genome analysis based on sequence typing

The genome assemblies had average N50 of 0.75 Mb (median 1.05 Mb), with between 7 and 69 contigs (median 13) per isolate, consistent with high-quality draft assemblies suitable for downstream analyses (see Additional files 1 and 2). A total of 137 out of 138 isolates were successfully assigned unique Bacterial Isolate Genome Sequence Database (BIGSdb) identities (IDs); one isolate (Bac ID 35) is pending allele number assignment. Among the *S. uberis* isolates, 68 (49.3%) were assigned an ST, and 49 (35.5%) were assigned a cgST. In total, six STs were identified. Two isolates belonged to ST241 (farm B, Bac IDs 2 and 15) and the remaining 135 isolates (98.5% of 137) represented Thailand-unique sequence types (STs), namely ST2048, ST645, ST647, ST651, and ST655. The most prevalent ST was ST2048 (*n* = 27 isolates, farm A = 7, farm B = 20), followed by ST645 (*n* = 23 isolates, from farm A = 22, farm B = 1), ST647 (*n* = 10 isolates from farm B), ST651 (*n* = 5 isolates from farm B), ST241 (*n* = 2 isolates from farm B), and ST655 (*n* = 1 isolate from farm B). The most prevalent cgST was cgST1258 (*n* = 3 isolates from farm A), with the remaining cgSTs identified in two or fewer isolates. It was not possible to assign STs to 50 isolates from farm C because of missing allele numbers for *gki, tdk, tpi,* or *yqiL*. The remaining six isolates from farm C were assigned to cgST1285–cgST1289, with cgST1288 occurring in two isolates (Bac IDs 103 and 104).

**Figure 1:**
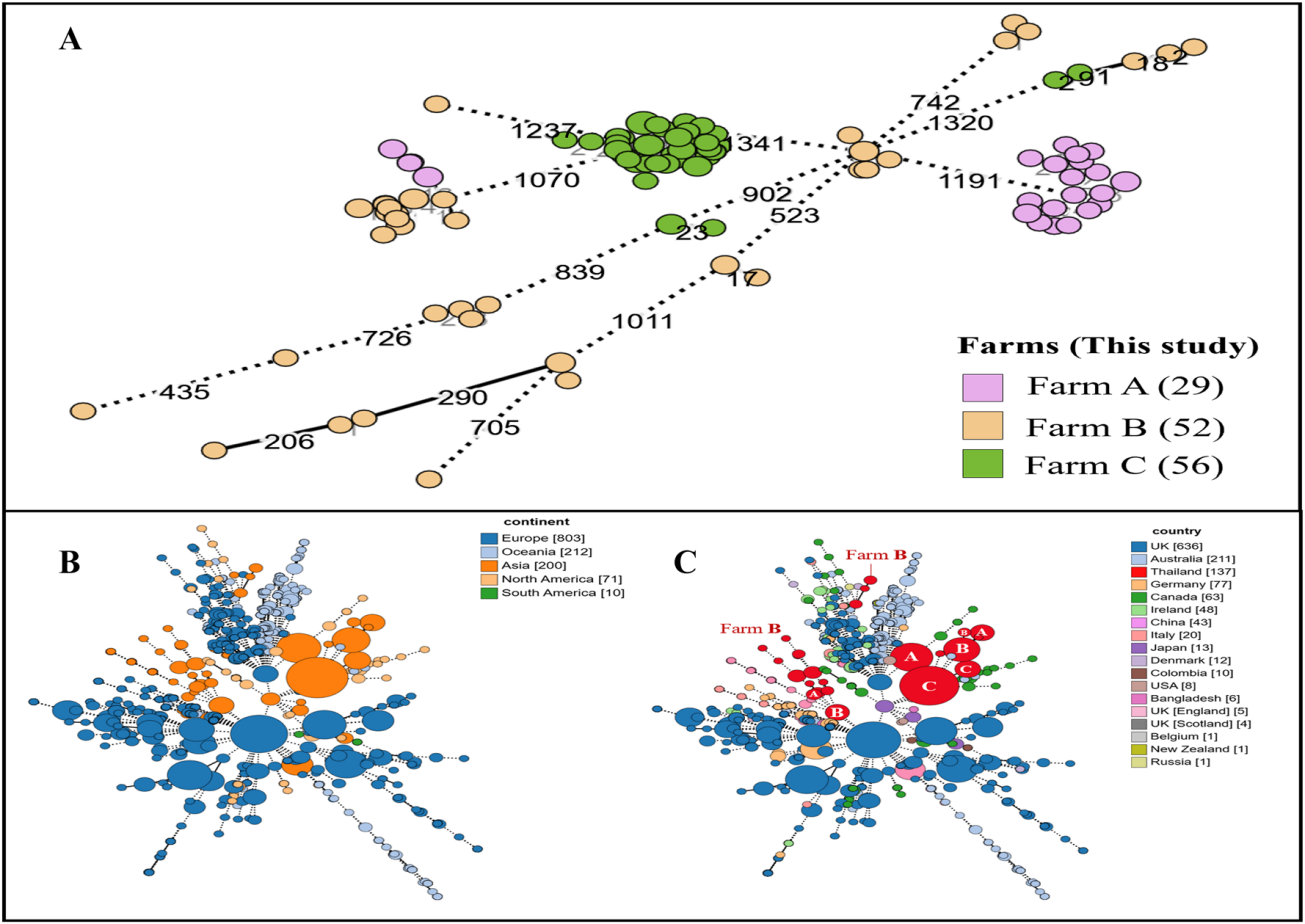
**Minimum spanning tree (MST) based on core genome multi-locus sequence typing (cgMLST) profiles of** ***S. uberis* genome sequences, including 137 isolates from Thailand and 1251 isolates from other countries**. **A** Minimum spanning tree of *S. uberis* isolates obtained in this study. The colors indicate the three farms: farm A (pink), farm B (orange), and farm C (green). Nodes are connected by solid or dashed lines, with numbers indicating allelic differences. Solid lines denote close genetic relatedness, whereas dashed lines represent more distant relationships. **B** The global cgMLST MST for all *S. uberis* genomes from the PubMLST database, colored by continent. **C** The global tree with Thai isolates overlaid and highlighted in red, showing the placement of farm A, B, and C *S. uberis* isolates within the global *S. uberis* population structure

The relationships between the *S. uberis* isolates from this study and those reported globally in the *S. uberis* pubMLST database are illustrated in Figure 1. Both the cgMLST and MLST schemes, comprising 1812 and 7 loci, respectively, revealed a phylogenetic structure in which Thai *S. uberis* grouped predominantly according to their farm of origin (Figure 1A). When integrated into the global dataset, the topology showed clear geographic clustering of international *S. uberis* (Figure 1B). Thai *S. uberis* were dispersed across multiple global lineages, which also included isolates from Japan, China, Australia, Canada, and the UK (Figure 1C).

**Figure 2:**
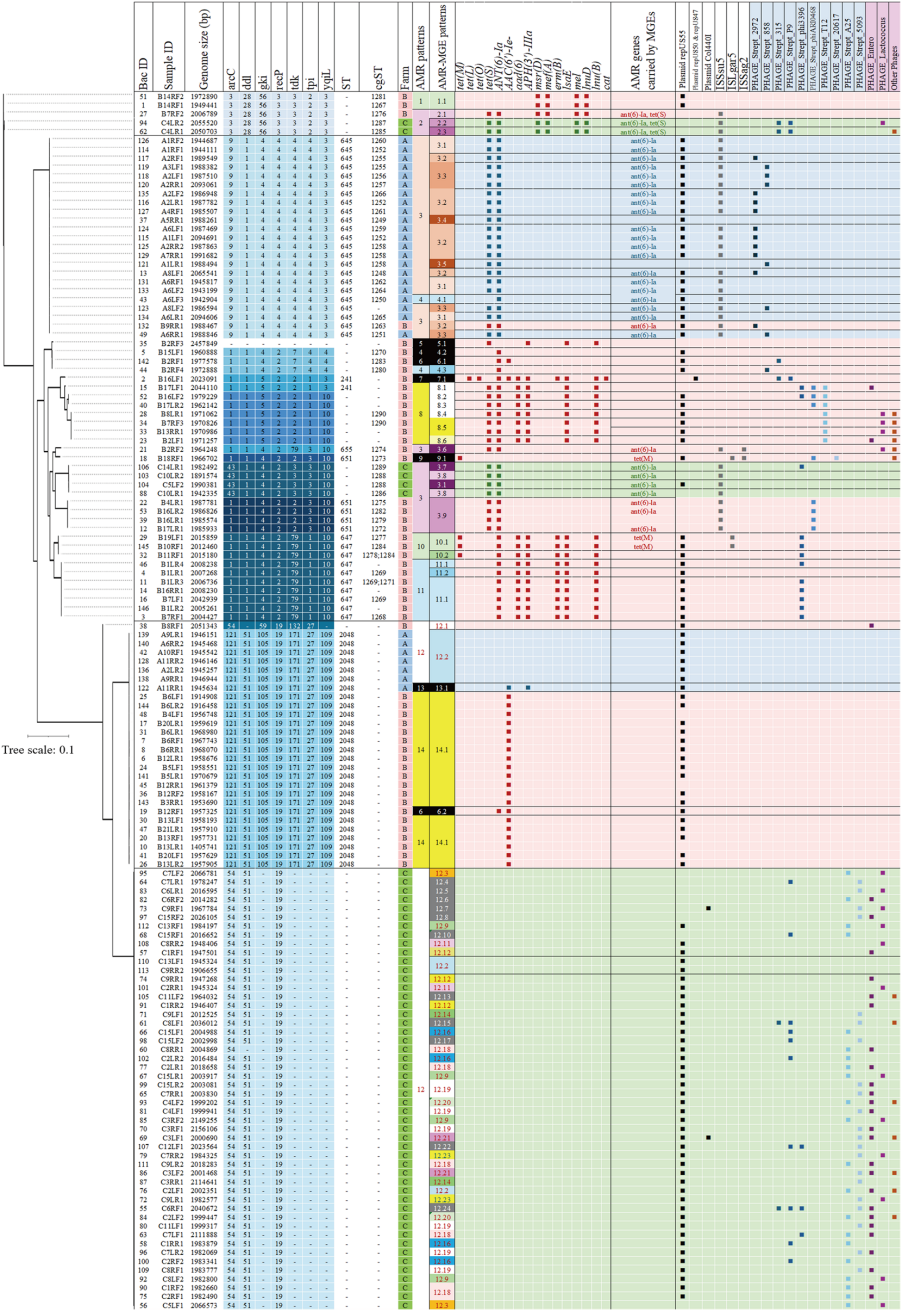
**Core-genome single-nucleotide polymorphism-based phylogenetic tree of 138 mastitis-associated** ***S. uberis * isolates from Thailand**. The phylogenetic tree was constructed using Roary and visualized via iTOL. The scale bar indicates 0.1 substitutions per nucleotide position, with 100 bootstrap replicates. The Bac ID, Sample ID, allelic profiles, sequence type (ST), core genome ST (cgST), antimicrobial resistance (AMR) genes, and mobile genetic elements (MGEs), where assigned, are indicated. In the Sample ID column, each code represents farm, cow, quarter, and sampling time in the format farm-cow-quarter-sampling time, where *RF* right front, *LF* left front, *RR* right rear, and *LR* left rear. The phylogenetic and AMR data were obtained from the study of Srithanasuwan [7]

The phylogenetic analysis of the *S. uberis* population revealed a structured population composed of several clades separated by deep branches (Figures 1 and 2). Within each major clade, isolates formed multiple subclades with short branch lengths, consistent with recent clonal expansion. Sequence typing results were largely consistent with the phylogeny of the *S. uberis* population [7]. For example, ST645 and ST2048 corresponded to two monophyletic clusters that were both found across farms A and B (Figure 2), with additional ST in farm B corresponding to additional branches. The only exception was ST651, which was found on two branches in herd B.

Simpson’s index of discrimination (SID) based on STs varied among farms, with farm B showing the highest discrimination (SID = 0.759) and farm A showing moderate discrimination (SID = 0.431). For farm C, ST assignment was not possible for all isolates, resulting in an apparent SID of 0, which reflects lack of typeability rather than true lack of diversity. The discriminatory index based on AMR gene patterns was generally greater than that based on STs, with farm B exhibiting considerable heterogeneity (SID = 0.830), whereas farm C, which had lower AMU, remained relatively uniform (SID = 0.200). Farm A showed moderate discrimination based on AMR genes alone (SID = 0.446). By contrast, discrimination based on AMR–MGE patterns was high across all farms (SID = 0.833–0.956), with farm C showing the highest value (SID = 0.956), indicating considerable variation driven by the distribution of MGEs despite low core genome heterogeneity.

Epidemiological information, including farm, cow, quarter, and sampling time, was encoded in the Sample ID column as shown in Figure 2 to capture epidemiological dynamics and clonal relationships. In longitudinal data, both strain replacement (different core genome) and strain evolution (same core genome with different accessory genome) were observed. For instance, isolates indicated with Sample ID C5LF1 and C5LF2 both originated from the left-front quarter of cow no. 5 on farm C in 2023, but they belonged to separate branches of the phylogenetic tree, and had distinct STs and MGE patterns, indicating either coinfection by two strains (only one isolate per sample was sequenced) or sequential episodes of IMI caused by different strains. Evolution of the accessory genome during IMI is illustrated by isolates C4LR1 and C4LR2 (left rear quarter of cow no. 4 in farm C), which had identical STs and carried identical AMR genes but different prophages.

### Recombination analysis

ClonalFrameML analysis revealed distinct recombination dynamics among *S. uberis* isolates from these three farms, with farm B showing the highest recombination-to-mutation ratio (*R*/*θ* = 4.42), compared with farm A (0.00007) and farm C (0.00294), as shown in Table 1. This indicated that in farm B, recombination contributed significantly more to genetic diversification than point mutation. The divergence of the imported DNA in farm B (D = 0.238) also coincided with greater genomic diversity, a wider range of MGEs, and a broader spectrum of AMR genes (see below). The average recombined fragment size (*I*) varied. Farm A exhibited the longest tracts (~1000 bp) but with extremely low recombination frequency. By contrast, farm B had shorter fragments or tract lengths (1/*δ* = 0.0337), consistent with frequent gene exchange within a closely related local pool. farm C displayed low recombination frequency but maintained high MGE diversity, suggesting that MGEs played a larger role than chromosomal recombination in shaping its genetic variation.

**Table 1:** **Recombination parameters estimated by ClonalFrameML.**

Parameters	Farm A	Farm B	Farm C
Recombination-to-mutation ratio	0.00007	4.42	0.00294
The average size of recombined fragments (bp)	1000	30	87
The inverse of the mean recombination tract length	0.001	0.0337	0.0115
The divergence of imported DNA	0.100	0.238	0.031

**Figure 3:**
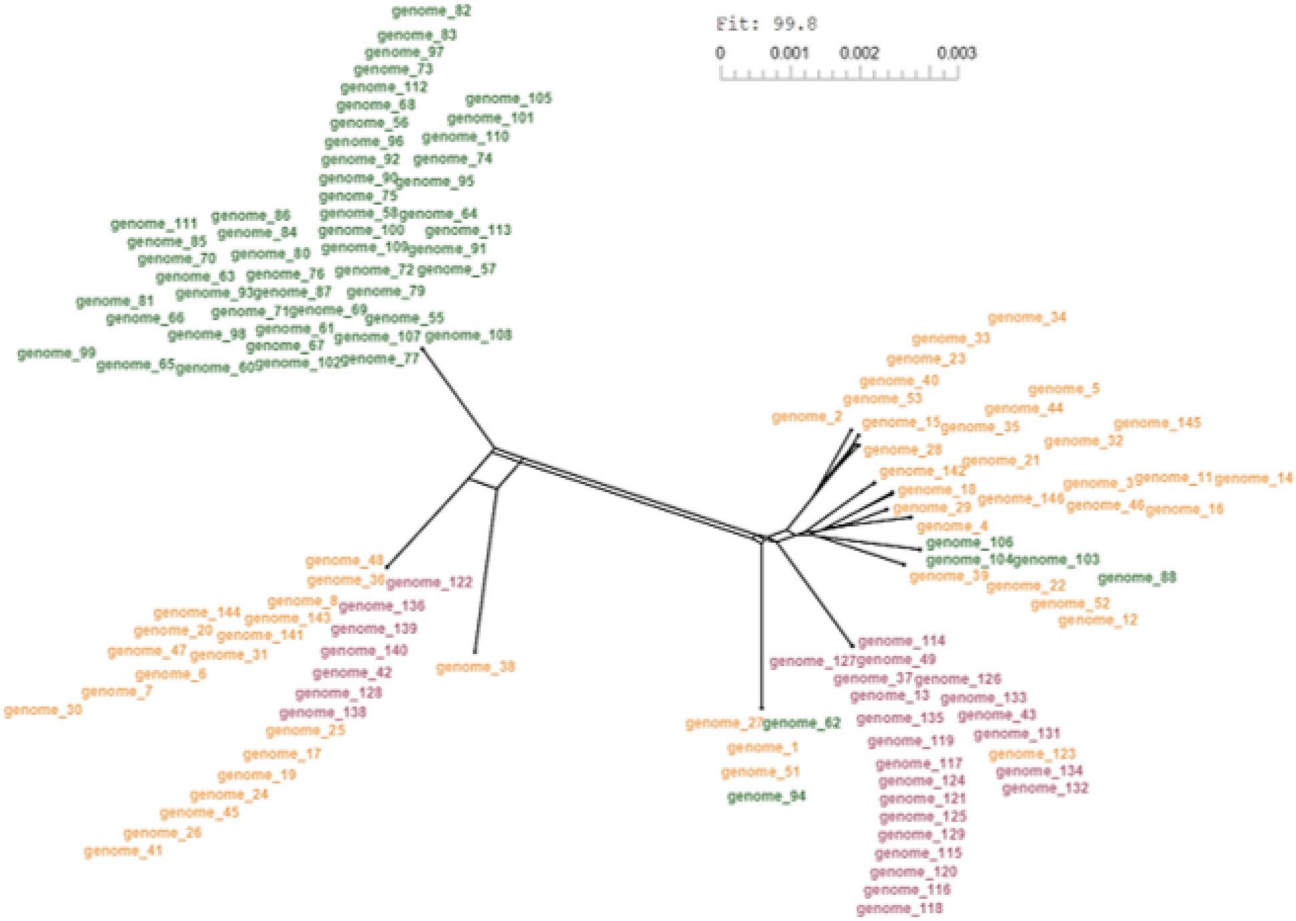
**Neighbor-net network constructed in SplitsTree on the basis of core genome single nucleotide polymorphisms of** ***S. uberis* isolates from three dairy farms in Thailand**. Each genome number represents the corresponding Bac ID. Colors indicate farm of origin and year of sampling: farm A (purple, 2013), farm B (orange, 2019), and farm C (green, 2023). Reticulate connections represent conflicting phylogenetic signals consistent with recombination or horizontal gene transfer

Neighbor-net analysis supported the ClonalFrameML findings by visualizing the genomic relationships and recombination patterns among the *S. uberis* isolates (Figure 3). Overall, a low level of recombination was detected in this study. Reticulate connections in the central region of the network indicated potential recombination events or horizontal gene transfer in the core genome, particularly among isolates from farm B, which is consistent with the higher recombination rates observed in *S. uberis* populations in this farm. By contrast, the farm C isolates formed a tight, coherent cluster with little internal branching, apart from six genomes that clustered with *S. uberis* genomes from farm B on two distinct branches. The lowest level of recombination was observed in farm A, where the majority of genomes for a single cluster, with seven genomes grouped in a second, distinct cluster with minimal evidence of network reticulation.

### Mobile genetic elements

Three insertion sequences (IS) were detected among the isolates. Most *S. uberis* isolates (*n* = 34) harbored a single IS, either ISSsu5 (*n* = 32) or ISLgar5 (*n* = 2). Two *S. uberis* isolates carried two distinct IS elements, with BacID 17 possessing ISSag2 and ISLgar5, while BacID 20 carried ISSsu5 and ISSag2. Overall, 35 of 138 *S. uberis* isolates carried AMR genes colocated on the same contig with MGEs, particularly insertion sequences. The majority of isolates carried plasmid-associated genes, notably repUS55, repUS50 and repUS47 [7].

In farm A, 20 *S. uberis* isolates harbored ISSsu5 which was consistently colocated on the same contig with ANT(6)-Ia. In farm B, six and one isolate(s) carried ISSsu5 and ISSag2, respectively, colocated on the same contig with ANT(6)-Ia. Three and one isolate(s) carried ISLgar5 and ISSag2, respectively colocated with tet(M). Additionally, for isolates in farm C, 39 of 41 isolates carried repUS55, while 2 isolates harbored Col440I. Two isolates carried ISSsu5 colocated with both ANT(6)-Ia and tet(S), whereas four isolates carried ISSsu5 colocated with only ANT(6)-Ia.

Phage analysis revealed that 16 (55%), 27 (51%), and 51 (91%) *S. uberis* isolates from farms A, B, and C, respectively, carried prophages. An example of prophage regions carried by *S. uberis* genomes is shown in Additional file 3. Isolates from farm A harbored only two phage types: PHAGE_Strept_2972 (*n* = 10) and PHAGE_Strept_858 (*n* = 6). These two prophages were rarely detected in isolates from farms B and C, despite a greater diversity of prophages in isolates from those farms. In farm B, 11 types of prophages were detected in 27 isolates. PHAGE_Strept_phi3396 was the most frequently detected prophage in this farm (*n* = 11). In farm C, 10 distinct prophage types were detected despite the low SID in farm C, where isolates share a single core genome and AMR profile. This suggested that even within an apparently homogeneous bacterial population, prophages may contribute to genomic variation through different configurations across isolates. Two phages, PHAGE_Strept_5093 and PHAGE_Strept_A25 were detected only in *S. uberis* strains without AMR genes from farm C, showing that selection for AMR is not the only evolutionary driver of horizontal gene transfer (HGT). Additional file 4 provides details of the prophages identified in each isolate.

**Figure 4:**
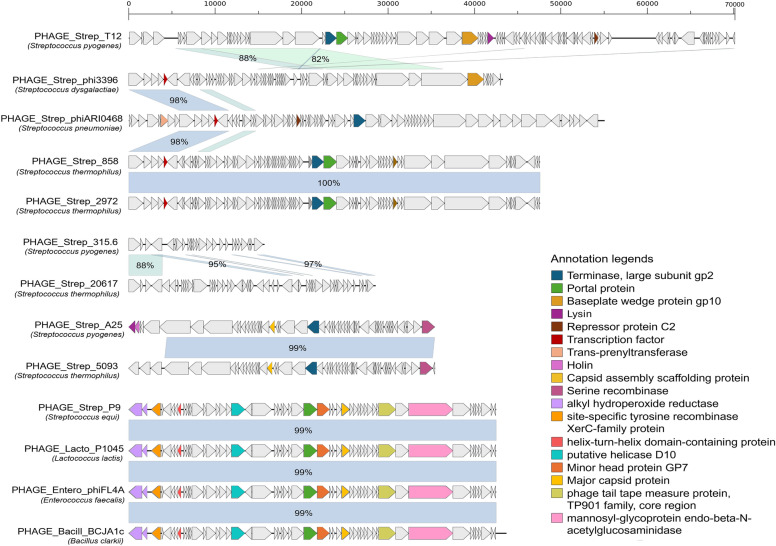
**Syntenic analysis between prophages detected in** ***S. uberis*** **and matching prophages from other bacterial genera**. Linear genome maps of prophages are displayed, with coding DNA sequences (CDSs) represented as arrows indicating transcriptional orientation. Colored arrows denote genes with known or putative functions, as described in the annotation legend. Prophage names and their corresponding original host species are indicated on the left. BLASTN-based alignments (nucleotide identity >80%) were generated and visualized using GenoFigure Blue connecting regions between genomes indicate orthologous nucleotide sequences sharing the same orientation

Alignment of prophages (Figure 4) showed a conserved, syntenic organization between the prophage sequences both within and between genera. All prophages were identified in *S. uberis* genomes; however, database annotations indicate that closely related prophages were originally described in other *Streptococcus* species, including *S. pyogenes*, *S. dysgalactiae*, *S. pneumoniae*, and *S. thermophilus*. Prophages infecting *S. pyogenes* (PHAGE_Strept_A25) and *S. thermophilus* (PHAGE_Strept_5093) exhibited very high sequence similarity (99%) to each other. Additionally, PHAGE_Strept_858 showed 100% nucleotide identity with PHAGE_Strept_2972. Both are prophages of *S. thermophilus* and were detected in *S. uberis* isolates from farm A.

Sharing between genera was observed, as a prophage in our *S. uberis* collection, which has also been described in *S. equi* (PHAGE_Strept_P9), showed high sequence similarity to prophages identified in other gram-negative, catalase-positive species that may cause mastitis, i.e., *Lactococcus lactis* (PHAGE_Lacto_P1045) and *Enterococcus faecalis* (PHAGE_phiFL4A), as well as a *Bacillus clarkii* prophage (PHAGE_Bacilli_BCJA1c). Over 99% nucleotide identity was detected between their structural genes such as those encoding DNA replication, head capsid, tail genes, and terminase subunits. Although most annotated proteins corresponded to typical phage structural and regulatory modules, some host-like enzymes (e.g., trans-prenyltransferase, mannosyl-glycoprotein endo-β-N-acetylglucosaminidase, and alkylhydroperoxide reductase) were also identified, both in phages infecting *S. uberis* and in those infecting other genera.

## Discussion

This study aimed to explore the contribution of recombination and MGEs to *S. uberis* adaptation and evolution across diverse population structures. Results demonstrated a high prevalence of MGEs with frequencies varying across farms. Similar enrichment with mobile and exogenous DNA has also been detected in other members of the Pyogenes group of streptococci, notably in Group A and Group B Streptococcus [30, 31]. These MGEs are involved in the bacteria's ability to adapt to selective pressures, including AMU [32], and numerous putative and proven resistance genes are located on MGEs [33, 34].

Consistent with previous studies reporting a positive association between AMR gene abundance and MGE prevalence and diversity [35, 36], genome annotation of Thai *S. uberis* isolates revealed frequent colocation of AMR genes with MGEs. Previous work on the same isolate collection has shown that AMR is strongly associated with farm-specific antimicrobial usage (AMU) policies and drug regulation practices [7]. Together, these findings suggest a link between AMU, AMR, and MGEs, with MGEs playing a central role in the dissemination and persistence of resistance determinants within the population. In particular, insertion sequences (IS) were commonly detected adjacent to resistance determinants. IS elements, often referred to as “jumping genes,” can autonomously move within the genome and facilitate the mobilization of AMR genes [37]. In this study, the ISSsu5 and ISLgar5 elements were found adjacent to the aminoglycoside resistance gene ANT(6)-Ia and the tetracycline resistance gene tet(M), respectively. Similar associations between IS elements and AMR genes have been widely reported, underscoring their role in shaping resistance phenotypes and highlighting their importance for predicting and addressing future resistance determinants [38, 39].

Consistent with our findings, large-scale genomic studies have demonstrated that *S. uberis* populations exhibit weak geographic structuring, with isolates from different continents interspersed across the phylogeny rather than forming distinct regional lineages [40]. In line with this, our Thai isolates do not form a single distinct cluster but are distributed among multiple lineages shared with international isolates (Figure 1C). Similarly, *S. uberis* populations worldwide display extensive genetic heterogeneity, largely driven by high levels of accessory genome variation [40]. Comparable patterns have been reported in studies from the Czech Republic [41], Germany [42], and Australia [43].

In addition, results reveal a remarkably dynamic accessory genome, predominantly driven by MGEs, even among epidemiologically linked isolates. Despite the relatively low recombination rates and the conserved core genome observed in this *S. uberis* population, a high degree of MGE diversity was observed among isolates obtained from the same farm, cow, and even from the same quarter over time. This suggests that in populations where the core genome remains stable, MGEs serve as the primary drivers of micro-evolution and short-term adaptation. These findings are supported by genomic investigations of other pathogens, such as community-associated MRSA outbreaks, which demonstrate that the majority of within-clone genomic variation arises from the rapid gain and loss of MGEs rather than core genome recombination [44]. This high accessory plasticity allows the pathogen to maintain a stable lineage while rapidly acquiring transient traits beneficial for survival within the host environment.

Interestingly, this study provides evidence of the genetic exchange involving multiple types of MGEs, including IS and prophages, both within and between bacterial species and genera. Notably, the most prevalent IS identified in our *S. uberis* isolates was ISSsu5, an element originally characterized in *S. suis* [45], followed by ISLgar5, which was first described in *Lactococcus garviae* according to ISFinder [46], and an insertion sequence from *S. agalactiae*, ISSag2. While *L. garviae* and *S. agalactiae* may co-occur with *S. uberis* on dairy farms, colocation of pigs, the main host of *S. suis*, and dairy cattle, the main host of *S. uberis*, is unusual in Thailand. Thus, transfer of an IS from *S. suis* may have taken place via an intermediate bacterial host species and/or an intermediate human or animal host species. The discovery of these ISs were also reported in other species such as Group A *Streptococcus* (GAS) [47] and *Staphylococcus aureus* [48], suggesting a broad host range and high mobility for these elements within and beyond the dairy niche.

Furthermore, several prophage regions exhibited high sequence identity to prophages known to infect other bacterial genera, including *L. lactis* and *E. faecalis*. Both genera can be found in the gastrointestinal (GI) tract of cattle and in the mammary gland as a cause of mastitis [49, 50]. Given that *S. uberis* also colonizes both the mammary gland and the GI tract [51], it may acquire accessory genome content from multiple genera in both niches, including via prophages [52]. Such genetic exchange among cocolonizing species has been well-documented for mastitis-causing streptococci [13, 53] and in other microbial ecosystems [54]. Ecological overlap between bacterial species may facilitate cross-genus genetic exchange, suggesting a potential role of microbial ecology in the evolution and diversification of *S. uberis*. In the current analysis, some prophages with 100% nucleotide identical sequences were reported under different prophage names. As a database-driven system, PHASTEST may assign different “top hits” or be limited to recognizing phages on the basis of homology to existing records, often resulting in identical sequences being given different names because of redundant or synonymous entries in the reference database [55]. Given the frequent occurrence of prophages in *S. uberis*, development of a dedicated typing scheme for phages, as has been described for *S. agalactiae* [18], may be worth considering.

Other limitations of the current study include the epidemiological origin of the material, which represented only three farms sampled in different years (2013, 2019, and 2023), as well as limitations of the databases used for genomic analyses. The three farms illustrate different management styles and bacterial population structures, but because each population structure is specific to one farm, we can not draw causal inferences about the relation between farm management, *S. uberis* population structure, and evolutionary mechanisms. The difference in sampling years represents a minor limitation, as it is difficult to clearly determine whether the observed differences are driven by temporal changes or farm-specific factors. Future studies incorporating longitudinal sampling or multiple farms within the same time frame would help to address this.

The marked difference in recombination estimates (*R*/*θ*) between farm A (0.00007) and farm B (4.42) is likely influenced by differences in population structure and levels of clonality between farms rather than reflecting true biological differences alone. This result should be interpreted with caution. Additionally, database limitations also affected both core- and accessory-genome analysis. In particular, MLST and cgMLST could not assign allelic profiles to all isolates, as previously reported for *S. uberis* [10, 56] and other mastitis pathogens, such as *S. agalactiae* [57]. Likewise, standard MGE-detection tools may not have identified all species-specific MGEs. It should be noted that this study focused on the detection of acquired resistance genes and MGEs. Chromosomal mutations, such as those affecting penicillin-binding proteins, which may also contribute to antimicrobial resistance [58], were not investigated. Future studies employing targeted strategies could better identify MGEs unique to *S. uberis*, providing deeper insights into their roles in bacterial adaptation and AMR.

## Conclusions

This study reveals recombination events and MGEs associated with AMR genes in *S. uberis* isolated from different farm environments. Isolates from the farm with a higher prevalence of AMR genes were also characterized by frequent detection of MGEs colocated with these genes, with IS showing a strong association with AMR determinants. Despite the low recombination rates and the conserved core genome observed in the *S. uberis* populations in farms A and C, this study identified a high degree of MGE diversity. The data suggests ongoing local gene exchange both within and between bacterial genera. In particular, the homology between *S. uberis* IS and prophages, and those from other bacterial genera commonly present in dairy farm environments suggests that microbial ecology may influence the evolution of *S. uberis* through horizontal gene transfer. Overall, variation in phage diversity and MGE-mediated gene transmission across bacterial populations may play a significant role in evolution and short-term adaptation of *S. uberis*.

## Supplementary information

**Additional file 1. Genome assembly statistics and metadata of** ***Streptococcus uberis*** **isolates obtained from bovine mastitis cases**. Summary of the genomic assembly characteristics and associated metadata of *Streptococcus uberis* isolates collected from 3 dairy farms, including sample identifiers, host disease status, isolation source, BioProject and BioSample accession numbers, genome assembly size (total length), guanine–cytosinecontent, number of contigs, and N50 values.

**Additional file 2. Assembly statistics for** ***Streptococcus uberis*** **isolates used within this study**. Violin plots of key assembly statistics of all isolates used within this study. (A) The genome sizes of all isolates. (B) Percentage of GC bases in the genomes. (C) N50 of all isolates. (D) Number of contigs that make up the genome assemblies of each isolate. Violin plots were created using the R program, and genome statistics were determined using QUAST.

**Additional file 3. Representative circular genome maps of** ***Streptococcus uberis*** **isolates showing predicted prophage regions.** Representative circular genome maps of *Streptococcus uberis* isolates (Bac ID 2, Bac ID 3, Bac ID 12, and Bac ID 15) showing prophage regions predicted by PHASTEST. Prophage sequences are highlighted in green, while other coding sequences are annotated around the circular genome (accessed March 27, 2025).

**Additional file 4. Distribution of plasmids, antimicrobial resistance genes, and prophage-associated elements among** ***Streptococcus uberis*** **isolates**. Summary of plasmid replicons, antimicrobial resistance (AMR) genes, prophage regions, and prophage-associated genes identified in *Streptococcus uberis* isolates. The table includes the number of predicted prophage regions, total prophage-related genes, and the most frequently detected phage sequences based on gene hit counts.

## Data Availability

The whole-genome sequencing results of all *S. uberis* isolates are publicly available in the supplemental materials, the Sequence Read Archive database (https://www.ncbi.nlm.nih.gov/sra/docs/), and the National Center for Biotechnology Information GenBank (https://www.ncbi.nlm.nih.gov/datasets/genome/?taxon=1349) under BioProject accession number PRJNA883037.
