# Supplementary material for: Recombination, mobile genetic elements, and genetic transfer contribute to the adaptation of Streptococcus uberis causing mastitis
Source: Vet Res. 2026 Jul 7;57:127. doi: 10.1186/s13567-026-01795-x (PMC13343983; doi:10.1186/s13567-026-01795-x)
Supplement: Supplementary file 2 — Additional file 2. Assembly statistics for Streptococcus uberis isolates used within this study. Violin plots of key assembly statistics of all isolates used within this study. (A) The genome sizes of all isolates. (B) Percentage of GC bases in the genomes. (C) N50 of all isolates. (D) Number of contigs that make up the genome assemblies of each isolate. Violin plots were created using the R program, and genome statistics were determined using QUAST. [file 13567_2026_1795_MOESM2_ESM.docx]

SUPPORTING INFORMATION FOR

**Recombination, Mobile Genetic Elements and Genetic Transfer Contribute to the Adaptation of *Streptococcus uberis* Causing Mastitis**

A. Srithanasuwan, Y. Zou, R. N. Zadoks, W. Suriyasathaporn, and Y. H. Schukken


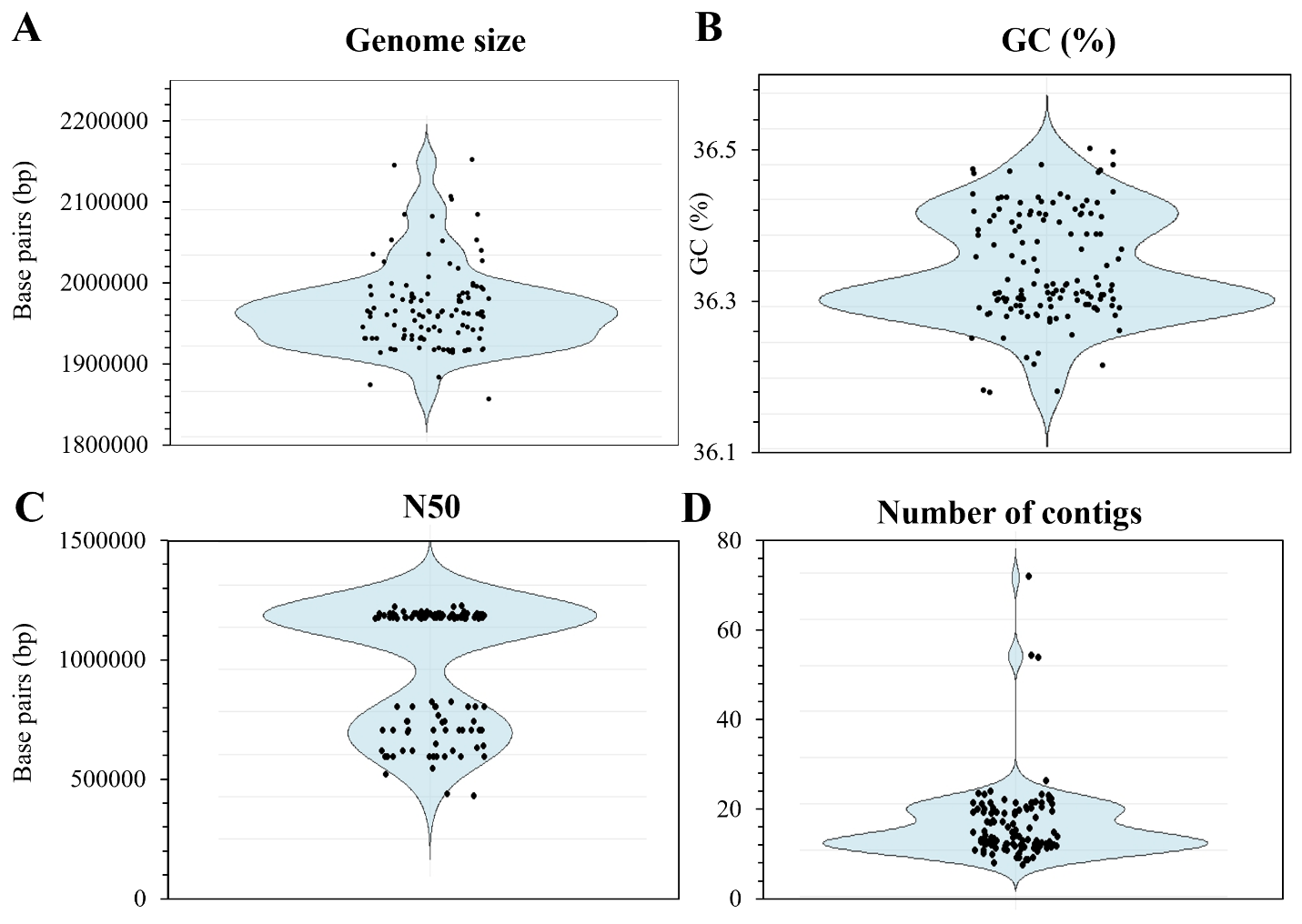
**Additional Figure** **Assembly statistics for *S. uberis* isolates used within this study.**

Violin plots of key assembly statistics of all isolates used within this study. (A) The genome sizes of all isolates. (B) Percentage of GC bases in the genomes. (C) N50 of all isolates. (D) Number of contigs that make up the genome assemblies of each isolate. Violin plots were created using the R program, and genome statistics were determined using QUAST.
