# Supplementary material for: Recombination, mobile genetic elements, and genetic transfer contribute to the adaptation of Streptococcus uberis causing mastitis
Source: Vet Res. 2026 Jul 7;57:127. doi: 10.1186/s13567-026-01795-x (PMC13343983; doi:10.1186/s13567-026-01795-x)
Supplement: Supplementary file 3 — Additional file 3. Representative circular genome maps of Streptococcus uberis isolates showing predicted prophage regions. Representative circular genome maps of Streptococcus uberis isolates (Bac ID 2, Bac ID 3, Bac ID 12, and Bac ID 15) showing prophage regions predicted by PHASTEST. Prophage sequences are highlighted in green, while other coding sequences are annotated around the circular genome (accessed March 27, 2025). [file 13567_2026_1795_MOESM3_ESM.docx]

SUPPORTING INFORMATION FOR

**Recombination, Mobile Genetic Elements and Genetic Transfer Contribute to the Adaptation of *Streptococcus uberis* Causing Mastitis**

A. Srithanasuwan, Y. Zou, R. N. Zadoks, W. Suriyasathaporn, and Y. H. Schukken


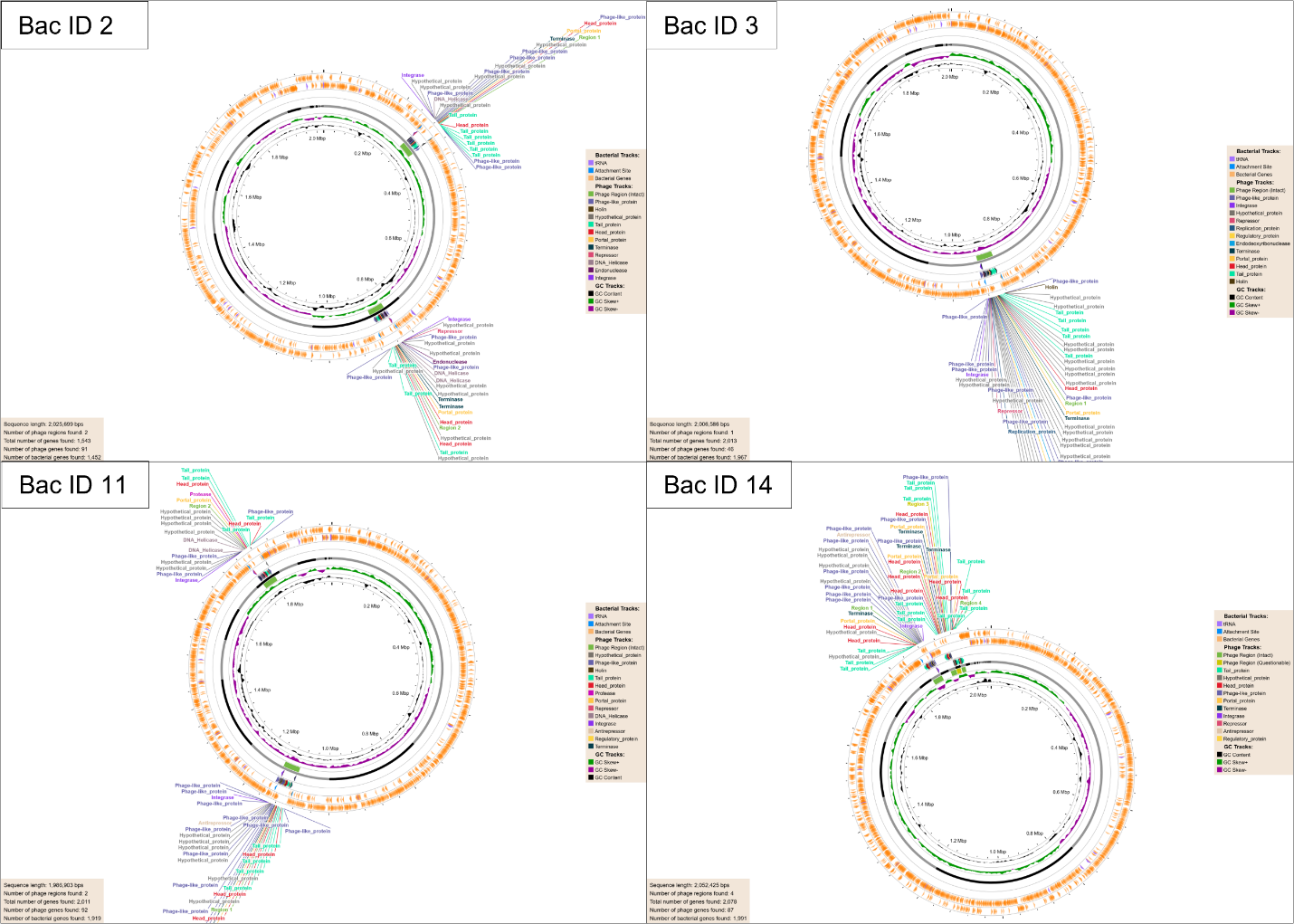


**Additional Figure Representative circular genome maps of Streptococcus uberis isolates showing predicted prophage regions**

Representative circular genome maps of *Streptococcus uberis* isolates (Bac ID 2, Bac ID 3, Bac ID 12, and Bac ID 15) showing prophage regions predicted by PHASTEST. Prophage sequences are highlighted in green, while other coding sequences are annotated around the circular genome. (accessed March 27, 2025)
